# Supplementary material for: Specific Attenuation of Purinergic Signaling during Bortezomib-Induced Peripheral Neuropathy In Vitro
Source: Int J Mol Sci. 2022 Mar 29;23(7):3734. doi: 10.3390/ijms23073734 (PMC8998302; doi:10.3390/ijms23073734)
Supplement: Supplementary file 1 [file ijms-23-03734-s001.zip › Holzer et al., 2022, Supplementary file S1.pdf]

Supplementary information for

Specific attenuation of purinergic signaling during bortezomib-induced peripheral neuropathy

Anna-Katharina Holzer, Ilinca Suciu, Christiaan Karreman, Thomas Goj, Marcel Leist

Table of Contents

	Page2 (P2)	Supplementary Methods
Table S1	P5	Schedule for differentiation media and supplementing factors
Table S2	P6	Primary antibodies used in this study
Table S3	P6	Agonists and antagonists used in Ca^{2+}-imaging experiment
Figure S1	P7	Characterization of iPSC-derived early-stage sensory neurons
Figure S2	P8	Exploration of toxicant responses (in the PeriTox test) of neurons generated and offered by a commercial supplier
Figure S3	P9	Time-dependency of compound effects assessed in the extended PeriTox test
Figure S4	P10	Variation of exposure time point and duration to assess compound toxicity
Figure S5	P11	Characterization of peripheral neurons derived by NGN1-overexpression
Figure S6	P12	Expression of functional TRPV1 channels
Figure S7	P13	Time dependent transcriptome profiling of PNN
Figure S8	P14	$[Ca^{2+}]_i$-dependent fluorescence signals of single cells
Figure S9	P15	Membrane depolarization and TRPV1 responses are unaltered upon toxicant exposure
Figure S10	P16	Reorganization of the cytoskeletal structure upon PI exposure
Figure S11	P18	Quantification of the nuclear area as cell viability indicator
Figure S12	P19	Reorganization of the microtubule structure as a general PI effect
	P21	Supplementary references

Supplementary Methods

Maintenance of induced pluripotent stem cells (iPSCs)

Maintenance of all iPSC lines used in this study was performed on human Laminin-521 (BioLamina, Sundbyger, Sweden) coating in essential 8 (E8) medium (Dulbecco's modified Eagle's medium/F12 [DMEM/F12] supplemented with 15 mM Hepes [Thermo Fisher Scientific, Waltham, MA, USA], 10 µg/ml holo-transferrin, 20 µg/ml insulin, 16 mg/ml L-ascorbic-acid, 0.7 mg/ml sodium selenite [all from Merck, Darmstadt, Germany], 100 ng/ml bFGF [Thermo Fisher Scientific, Waltham, MA, USA], 1.74 ng/ml TGFb [Bio-Techne, Minneapolis, MN, USA]) essentially as described [1]. Passaging of the iPSCs was performed every 7 days. Cells were incubated with EDTA (Thermo Fisher Scientific, Waltham, MA, USA) for 2 min (37°C, 5% CO_2) to detach the cells, so that clumps remain (no single cell suspension). IPSCs were washed off the plate with DMEM/F12. Cells were re-seeded in E8 medium on freshly coated plates in a final dilution of 1:40-60.

Differentiation of sensory neurons from iPSC

The iPSCs were prepared for neural differentiation on day of differentiation minus 2 (DoD-2) by replating in a single cell suspension (90,000 cells/cm^2) onto Matrigel^{TM} (Corning, Glendale, AZ, USA) coated 6-well plates in E8 medium supplemented with 10 µM Rock inhibitor (Y-27632 [Bio-Techne, Minneapolis, MN, USA]).
On DoD0', E8 was replaced by neural differentiation medium KSR (knock out DMEM with 15% serum replacement, 1 x Glutamax, 1 x nonessential amino acids, and 50 µM β-mercaptoethanol [all from Thermo Fisher Scientific, Waltham, MA, USA]) and the combination of five small molecule pathway inhibitors as described in detail in table S1. From DoD0'-5', Noggin and SB-431642 (both from Bio-Techne, Minneapolis, MN, USA) (± dorsomorphine hydrochloride [Dorso; Merck, Darmstadt, Germany]) were added, and CHIR99021 (Axon Medchem, Groningen, Netherlands), SU5402 (Bio-Techne, Minneapolis, MN, USA) and DAPT (γ-Secretase inhibitor IX; Merck, Darmstadt, Germany) were added on DoD2'-9'. From DoD4' onwards, KSR medium was gradually replaced by N2-S medium (DMEM/F12, 1 x GlutaMax [both from Thermo Fisher Scientific, Waltham, MA, USA], 0.1 mg/ml apotransferrin, 1.55 mg/ml glucose, 25 µg/ml insulin, 20 nM progesterone, 100 µM putrescine and 30 nM selenium [all from Merck, Darmstadt, Germany]) in 25% increments. On DoD9' or DoD12' (depending on the iPSC line used, see table S1), the cells were cryopreserved in 90% fetal bovine serum (FBS) (Thermo Fisher Scientific, Waltham, MA, USA) and 10% dimethyl sulfoxide (DMSO; Merck, Darmstadt, Germany).
After thawing of the pre-differentiated cells, sensory neuron precursors were cultured in 25% KSR and 75% N2-S supplemented with CHIR99021 (1.5 µM), SU5402 (5 µM) and DAPT (5 µM). Cells were seeded at a density of 100,000 cells/cm^2 on Matrigel^{TM} coated plates. For further differentiation and maturation, half of the medium was changed on DoD1 and DoD2. With the fresh culture medium on DoD2, Matrigel was added to the cells at a final dilution of 1:80. On DoD3, medium was changed to N2-S medium supplemented with 12.5 ng/ml brain-derived neurotrophic factor (BDNF), 25 ng/ml glia-derived neurotrophic factor (GDNF) and 25 ng/ml nerve growth factor (NGF) (all from Bio-Techne, Minneapolis, MN, USA) and 2 µM cytarabin (AraC; Merck, Darmstadt, Germany). Half of the medium was changed on DoD4, 7

and 10 with further Matrigel addition on DoD10. On DoD14, medium was changed to maturation medium (N2-S supplemented with BDNF [12.5 ng/ml], GDNF and NGF [both 25 ng/ml]). Half medium exchanges were performed every three to four days. Matrigel was diluted in the culture medium at a final dilution of 1:80 every 10 days.

Assessment of neurite area and cell viability

Cells stained with calcein-AM and HOECHST-33342 (H-33342) (both from Merck, Darmstadt, Germany) were imaged automatically using an ArrayScan VTI HCS microscope (Thermo Fisher Scientific, Waltham, MA, USA). Images were analysed for neurite area and cell viability by an automated algorithm. H-33342 staining was used to identify the cell nuclei. The nuclear area was enlarged to define the somatic area, which was then substracted from the calcein stain. The remaining calcein-positive pixels were quantified to derive the neurite area. Data on cell viability was derived from the same images by checking each H-33342 stained object (cell) for a double stain with calcein. Double-positive cells were classified as viable, calcein-negative cells as dead [2].

Immunofluorescence staining and microscopy

Neurons, grown on glass coverslips coated with Matrigel^{TM}, were fixed with 4% paraformaldehyde at 4°C over night. All further steps were performed at room temperature. Paraformaldehyde was taken off and cells were washed (~1 min) with phosphate buffered saline (PBS) followed by permeabilization with 0.6% Triton X-100 in PBS for 7 min. Coverslips were washed (~1 min) with PBS and blocked for 1 h in PBS containing 5% FCS and 0.1% Triton X-100. Primary antibodies (see Tab. S2) were diluted in fresh blocking solution and applied for 1 h. Residual free primary antibodies are then washed off with PBS. Secondary antibodies and H-33342 are diluted in blocking solution and applied on the coverslips for 30 min. After washing with PBS, coverslips were placed upside-down on mounting medium on microscope slides.

Transcriptome data analysis

For data processing, the R package DESeq2 (v1.32.0) was used for quality control, normalization and differential gene expression (DGE) analysis [3]. The raw probe counts were normalized to total sample counts per million (CPM). No library size threshold was used; samples with replicate correlation (Pearson R) to group average below 0.8 were removed from the analysis. Prior to the DGE, the low-count genes (less than 3 samples above 5 CPM) were discarded. The Wald statistics test was used for significance evaluation of each differentiation stage against DoD1 or DoD7. Selection of the most significant differentially expressed genes (DEG) was done using a (Benjamini-Hochberg) p-adjusted maximum threshold of 0.05 and a $\log_2$(fold change) minimum threshold of 1.

Measurement of changes in intracellular Ca^{2+}-concentration $[Ca^{2+}]_i$

Immature sensory neurons were seeded on DoD0 in 96-well plates at a density of 100,000 cells/cm^2. They were differentiated as described above for up to 45 days. One hour before the measurement, neurons were loaded with Fluo-4 Direct^{TM} Calcium Assay Kit (Thermo Fisher Scientific, Waltham, MA, USA) and H-33342 by exchanging half of the medium with the

staining solution. Pre-incubation with e.g. receptor antagonists was done together with Fluo-4 loading. Test compounds were diluted in Hanks' Balanced Salt Solution (HBSS) (Thermo Fisher Scientific, Waltham, MA, USA). Agonists and antagonists used in this study are listed in detail in table S3.

Monitoring of $[Ca^{2+}]_i$ was performed using a VTI HCS microscope (Thermo Fisher Scientific, Waltham, MA, USA) equipped with an automated pipettor and an incubation chamber. Neurons were kept at 37°C and 5% CO_2 during the experiments. Images were taken as fast as possible for 45 s and test compounds were applied automatically 10 s after the first picture was taken. In a standard experiment with 4 stimuli applied to one well (e.g. negative control, P2X3 agonist, TRPV1 agonist, KCl), the cells were imaged 4 times for 45 s with one stimulus applied at a time. Subsequent stimuli applied to one well were separated by a minimum interval of 5 minutes.

The images were exported as .avi video files and analysed with the CaFFEE software [4]. In brief, the time point of peak fluorescence was identified. Fluorescence data for the ground state (F_0) and for the peak time point (F_1) were assessed automatically for all cells. The difference between the two fluorescence levels, $\Delta F=F_1-F_0$, was used for further data processing [5].

__Supplementary Table S1: Schedule for differentiation media and supplementing factors__

X: applies for all iPSC lines
X: applies for mc-iPSC
X: applies for SBAD2
X: applies for Si28-NGN1

DoD	Medium		Supplements						
	KSR	N2S	Noggin (17.5 ng/ml)	SB431542 (10 µM)	Dorso (600 nM)	CHIR99021 (1.5 µM)	SU5402 (5 µM)	DAPT (5 µM)	Dox (2 ng/ml)
0’	X		X	X	X				
1’	X		X	X	X				
2’	X		X	X	X	X	X	X	
3’	X		X	X	X	X	X	X	
4’	X (75%)	X (25%)	X	X	X	X	X	X	X
5’	X (50%)	X (50%)				X	X	X	X
6’	X (50%)	X (50%)				X	X	X	X
7’	X (25%)	X (75%)				X	X	X	X
8’	X (25%)	X (75%)				X	X	X	X
9’	X (25%)	X (75%)				X	X	X	
10’	X (25%)	X (75%)				X	X	X	
11’	X (25%)	X (75%)				X	X	X	

Supplementary Table S2: Primary antibodies used in this study

Target	Isotype	Dilution	Supplier	Catalogue number	Lot number
βIII-tubulin (polymerized)	mouse IgG1	1:1000	BioLegend	921001	B216535
Peripherin	mouse IgG2a	1:200	Santa Cruz	sc-377093	A0319
Ki67 (PE)	mouse IgG1	1:500	BD Pharmingen	556027	03428
ISL1	rabbit	1:200	Abcam	ab109517	
BRN3A	rabbit	1:200	Merck Millipore	5945	2069332
P2X3	rabbit	1:200	Novus	NB100-1654	402376
F-actin	Phalloidin-555	1:500	Molecular Probes	8953S	0003

Supplementary Table S3: Agonists and antagonists used in Ca^{2+}-imaging experiments

Compound	Solvent	Concentration [μM]	Supplier	Catalogue number
α,β-methylene ATP	water	1	Cayman Chemical	10008956
AF-353	DMSO	0.1	Cayman Chemical	23034
capsaicin	DMSO	1	Cayman Chemical	92350
capsazepine	DMSO	10	Cayman Chemical	10007518
KCl	water	40,000	Merck	P9541

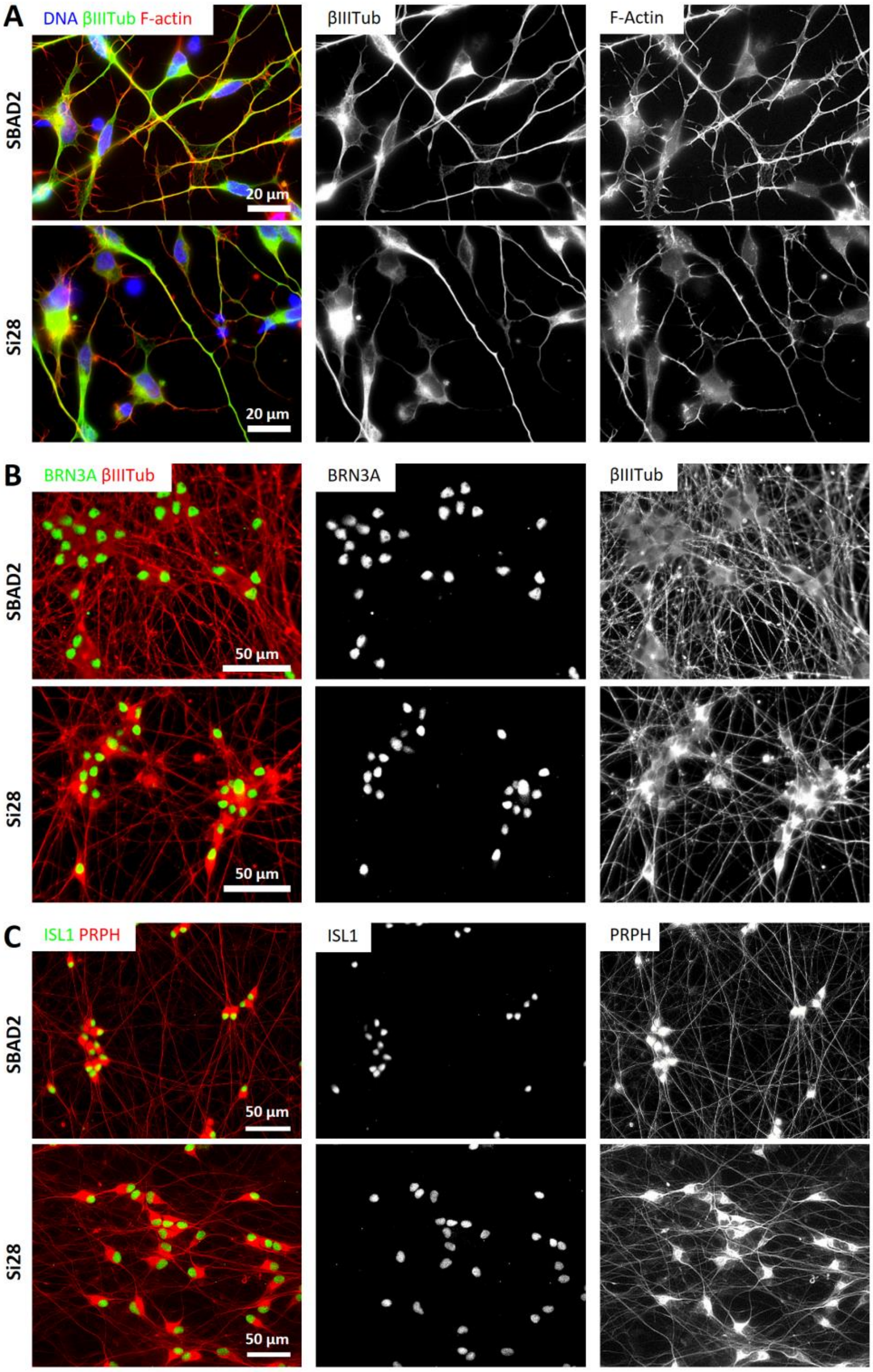

Fig. S1
Holzer et al. 2022

Supplementary Figure S1: Characterization of iPSC-derived early-stage sensory neurons

Neurons were differentiated from the iPSC-lines SBAD2 and Si28 according to the standard protocol . **(A)** Cells were fixed on DoD1 after thawing and stained for the pan-neuronal marker βIII-tubulin (βIIITub), and for F-actin. Growth cones with filopodia are evident on the growing neurites. **(B,C)** Cells were fixed on DoD7 and stained for βIIITub and the peripheral neuron transcription factors BRN3A **(B)** or Islet-1 and the intermediate filament peripherin (PRPH) **(C)**. Images of different fluorescent channels are shown to visualize the staining by individual antibodies. The composite images of the stainings are color coded according to the detail images (also see figure 1A). Scale bars are given in the images. All images are representative of typical cultures, but structures have not been quantified.

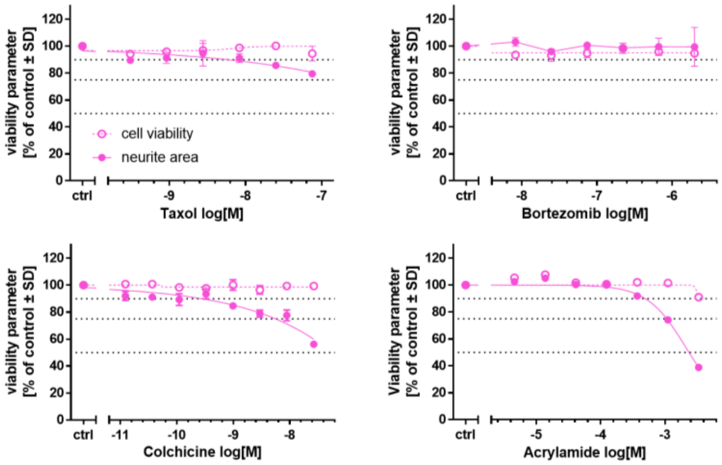

Fig. S2
Holzer et al. 2022

Supplementary Figure S2: Exploration of toxicant responses (in the PeriTox test) of neurons generated and offered by a commercial source.

The Peri.4U cell preparation, which had been generated to provide a commercial source of peripheral neurons, was used in the PeriTox test. Cells were exposed to the positive control compounds taxol, bortezomib, colchicine and acrylamide, which are known for their (peripheral) neurotoxic potential. Effects on neurite area (solid symbols and lines) and viability (open symbols, dashed lines) are shown. Data are means ± SD of 2 biological replicates.

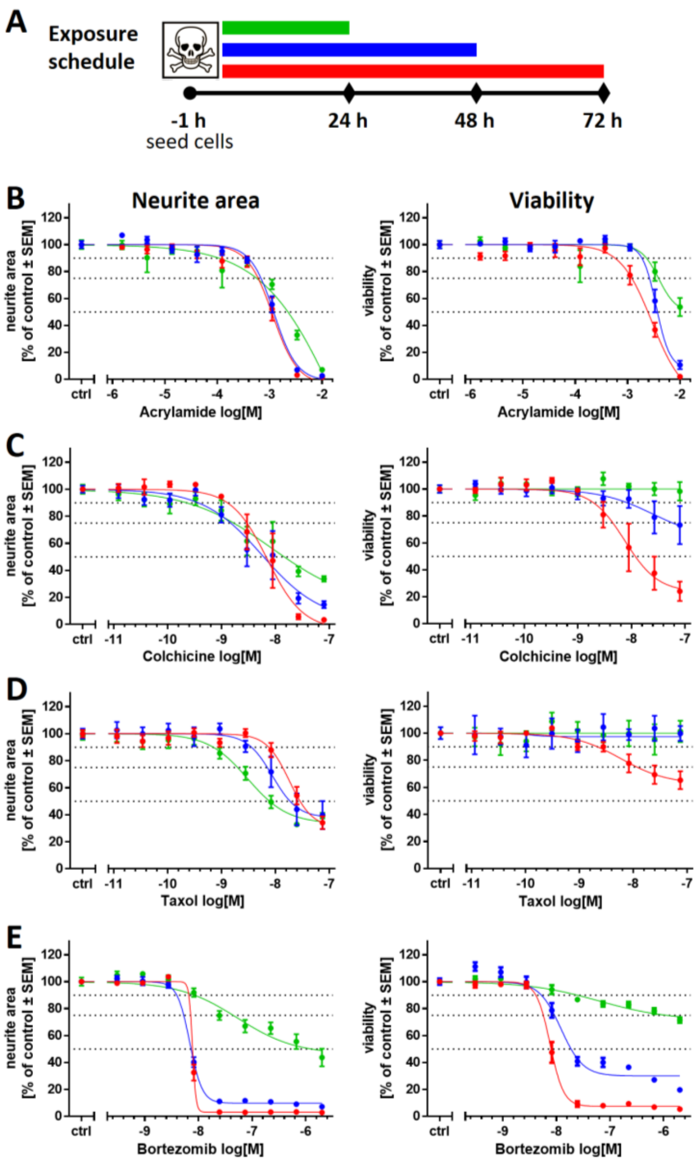

Fig. S3
Holzer et al. 2022

Supplementary Figure S3: Time-dependency of compound effects assessed in the extended PeriTox test

Compound-induced effects on the neurite area and cell viability were assessed in the extended PeriTox test. (**A**) Exposure schedules for the extended PeriTox test are depicted. One hour after plating of the peripheral neuron precursors, cells were exposed to the test compounds for 24 h (green), 48 h (blue) and 72 h (red). **(B-E)** Effects on neurite area (left) and viability (right) are shown for all three exposure times for the positive controls acrylamide **(B)**, colchicine **(C)**, taxol **(D)**, and bortezomib **(E)**. Data sets are colour coded to the respective exposure schedule in **(A)**. Data are means ± SEM of 2-3 biological replicates.

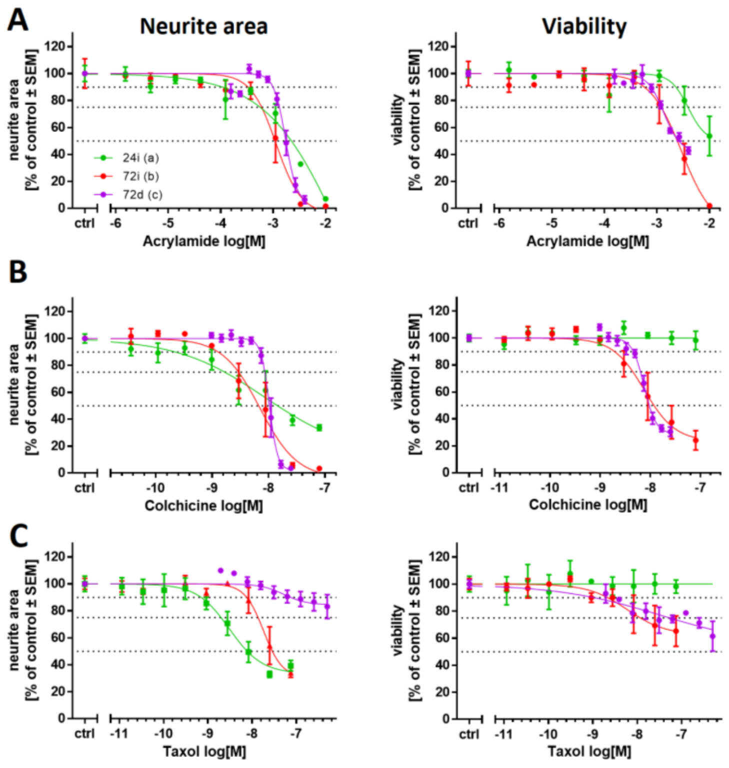

Fig. S4
Holzer et al. 2022

Supplementary Figure S4: Variation of exposure time point and duration to assess compound toxicity

The positive control compounds acrylamide **(A)**, colchicine **(B)** and taxol **(C)** were tested regarding their effects on immature peripheral neurons. Cells were exposed to the test compounds according to the exposure scenarios depicted in figure 2A: (a) immediately after thawing (1 h) for 24 h (green, 24i) and (b) 72 h (red, 72i), or (c) at a delayed time point, starting on DoD4, for 72h (purple, 72d). Compound effects on neurite area (left) and viability (right) are shown. Data are means ± SEM of 3 independent experiments.

.

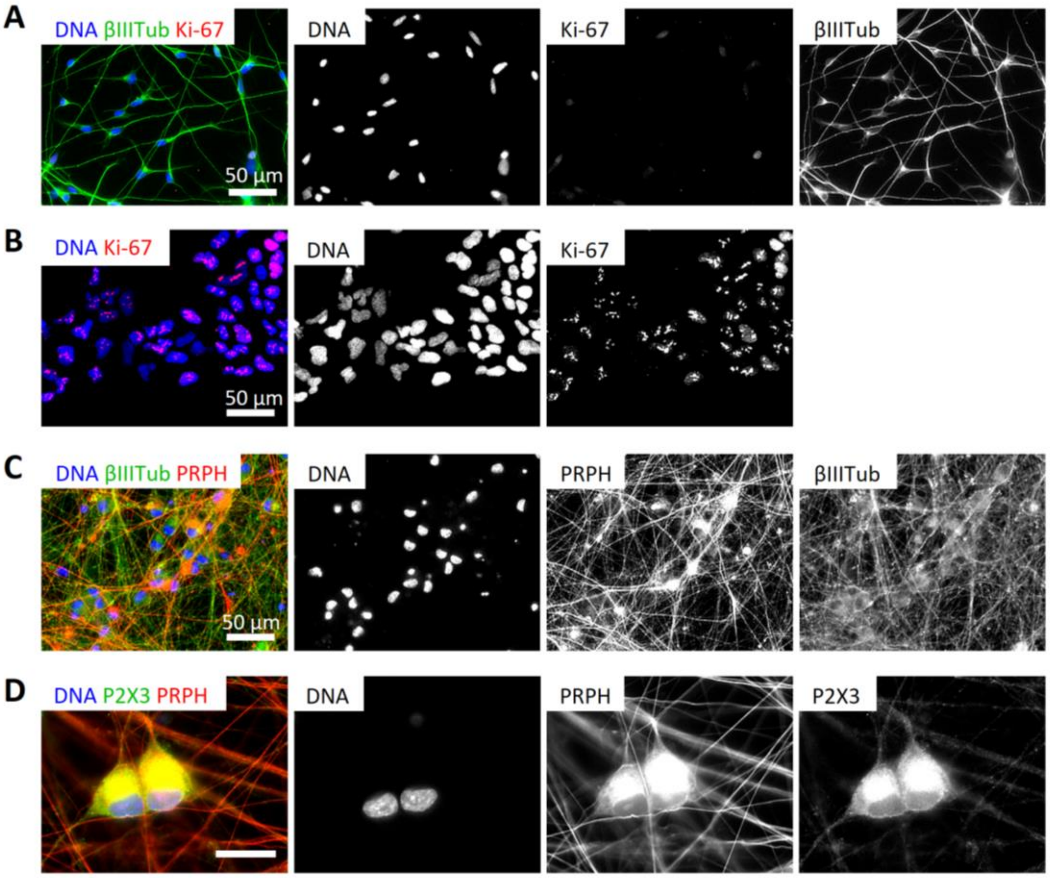

Fig. S5
Holzer et al. 2022

Supplementary Figure S5: Characterization of peripheral neurons derived by NGN1-overexpression

(A) Peripheral neurons differentiated from Si28-NGN1 iPSCs were fixed on DoD1 and stained for βIII-tubulin (βIIITub) and the proliferation marker Ki-67. **(B)** As a positive control for proliferating cells, neuroepithelial precursor cells [6] were fixed and stained for Ki-67, exhibiting the characteristic pattern of Ki-67-staining in the nucleus, thus verifying the functioning of the Ki-67-antibody. **(C,D)** Neurons were differentiated for 42 days after thawing and fixed. Immunofluorescence images show an extensive neurite network, stained positive for βIIITub and peripherin (PRPH) **(C)**, and the expression of P2X3 receptors in PRPH-positive cells **(D)**. Images of the single stainings are shown. The composite images of the stainings are color coded according to the detail images (**(A,C,D)** see also figure 3B). Scale bars are given in the images.

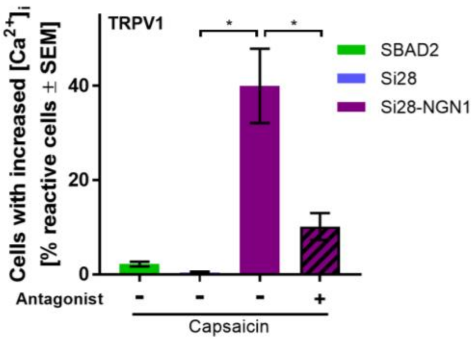

Fig. S6
Holzer et al. 2022

Supplementary Figure S6: Expression of functional TRPV1 channels

Peripheral neurons derived from the iPSC lines SBAD2 (green), Si28 (blue) and Si28-NGN1 (purple) were differentiated for >38 days and used for Ca^{2+}-imaging experiments. The TRPV1-specific agonist capsaicin [1 µM] was used to determine the expression of functional TRPV1 receptors. To confirm signaling through TRPV1, cells were pre-treated (1 h) with capsazepine, an antagonist of TRPV1, before stimulation with capsaicin. Data are means ± SEM of at least 3 biological replicates.

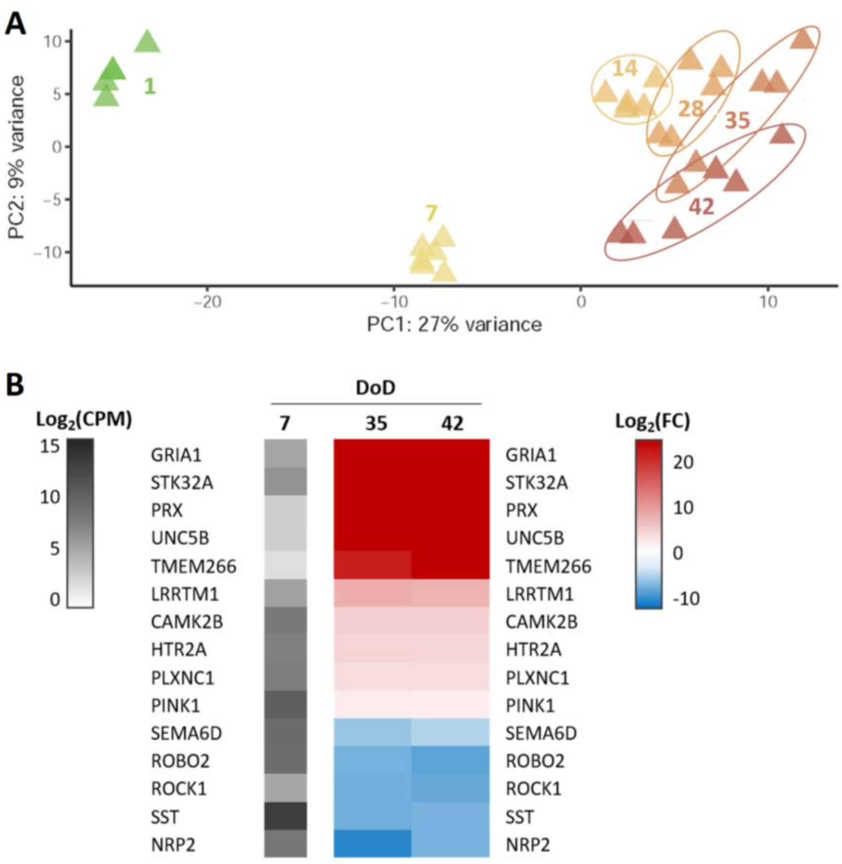

Fig. S7
Holzer et al. 2022

Supplementary Figure S7: Time-dependent transcriptome profiling of PNN

(A) PNN were grown from DoD1 - DoD42 and mRNA of different maturation stages was used for whole transcriptome analysis by TempO-Seq (full data in Supplementary file1, complementary data in figure 4). A PCA was performed for all of the >19,000 measured genes. In the two-dimensional PCA display, six maturation stages (day of differentiation (DoD) 1, 7, 14, 28, 35 and 42) of PNN are color-coded according to their DoD. Data points are derived from three independent differentiations, and all individual samples analyzed are depicted. **(B)** To gain insight into gene expression changes that occur during late differentiation, DoD35 and DoD42 samples were analyzed for differentially expressed genes (DEGs) *versus* DoD7. Examples of significant DEGs (adjusted *p*-value ≤ 0.05) with a fold change (FC) >4 were assembled. The left column shows the absolute expression levels of selected significant DEGs on DoD7 in counts of the corresponding gene per 1 million reads (CPM). The data for DoD35/DoD42 show the fold change (FC) of the expression levels *versus* DoD7. The color scales use $\log_2$ units (see supplementary files for complete data sets).

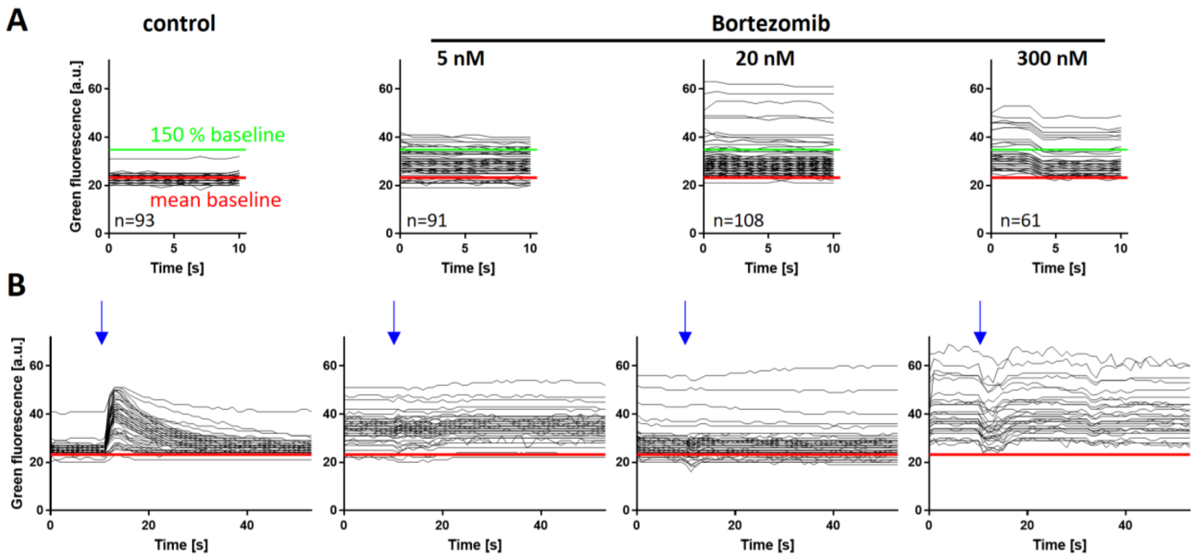

Fig. S8
Holzer et al. 2022

Supplementary Figure S8: $[Ca^{2+}]_i$-dependent fluorescence signals of single cells

Sensory neurons (>DoD38) were cultured under control conditions or exposed to bortezomib for 24 h, before Ca^{2+}-imaging was performed. Ca^{2+}-measurements depicted in **(A)** and **(B)** were executed with the same cells. **(A)** Baseline fluorescence of single sensory neurons recorded during the first 10 seconds of a Ca^{2+}-measurement, immediately before the first stimulus application. Fluorescence traces of one whole neuronal culture per pre-treatment condition with 61-108 single cells each are shown. Red horizontal lines indicate the mean baseline fluorescence of control cells. Green lines indicate the threshold of 150% control baseline fluorescence used for quantification of cells with increased resting cell $[Ca^{2+}]_i$ (also see figure S13). **(B)** Time-dependent fluorescence traces of single cells during experiments using the P2X3-specific agonist αβ-methylene ATP (addition to the cells is indicated by blue arrows). For clarity, only 50% of the cells depicted in **(A)** are shown (randomly chosen by software). Red horizontal lines correspond to the mean baseline fluorescence of control cells determined in **(A)**.

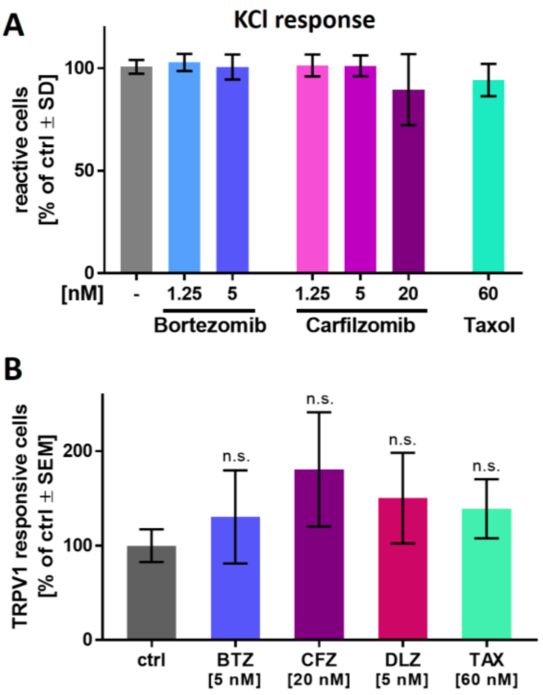

Supplementary Figure S9: Membrane depolarization and TRPV1 responses are unaltered upon toxicant exposure

Sensory neurons were differentiated for >38 days and used for Ca^{2+}-imaging experiments. Cells were pre-treated with the proteasome inhibitors bortezomib (BTZ), carfilzomib (CZF) and delanzomib (DLZ), or taxol for 24 h, before Ca^{2+}-measurements were performed. **(A)** Sensory neurons, which were first exposed to a P2X3-specific stimulus (see figure 5B,C), were subsequently subjected to an increased concentration of KCl [50 mM], which induces membrane depolarization in functional neuronal cells. Responsive cells were quantified. **(B)** Cells were stimulated with capsaicin [1 µM] and the number of responsive cells was assessed. Data are given as % of untreated control cells and are means ± SEM of at least 3 independent experiments. n.s., not significant

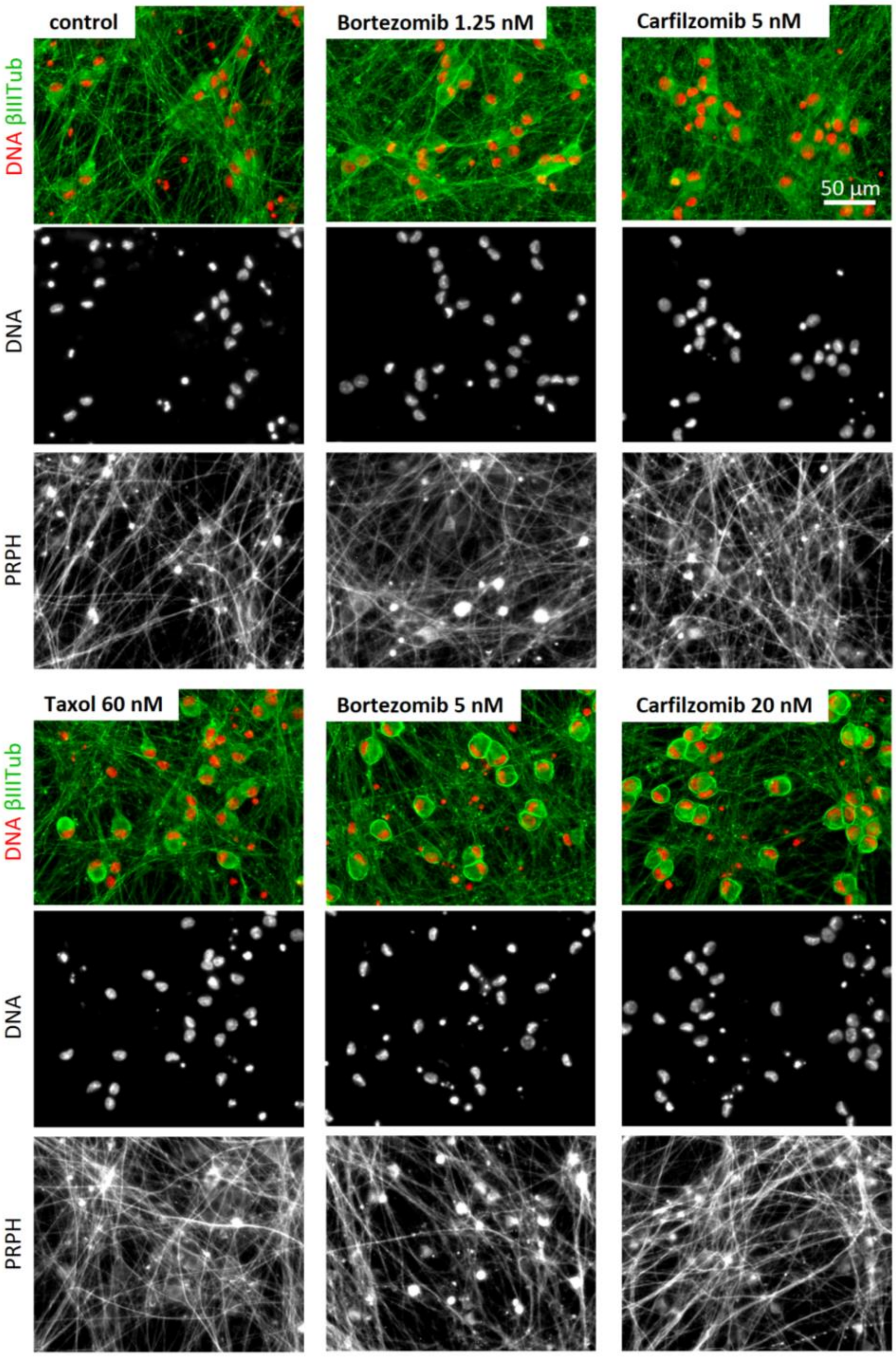

Fig. S10
Holzer et al. 2022

Supplementary Figure S10: Reorganization of the cytoskeletal structure upon PI exposure

Sensory neurons were differentiated for at least 38 days after thawing and exposed to bortezomib, carfilzomib or taxol for 24 h before fixation. Representative immunofluorescence images are shown (see also figure 6A). Composite images show stainings of nuclei (DNA, red) and βIII-tubulin (βIIITub, green), revealing aberrant staining for βIIITub at high concentrations of bortezomib or carfilzomib. Single staining images shown for (i) the nuclei (DNA) and (ii) the corresponding peripherin (PRPH) staining (for clarity not included in the composite images). Peripherin staining of PI-treated cells did not exhibit βIIITub-like reorganization to rings, but rather a clustering in the soma (outside the nucleus). The scale bar is given in the images.

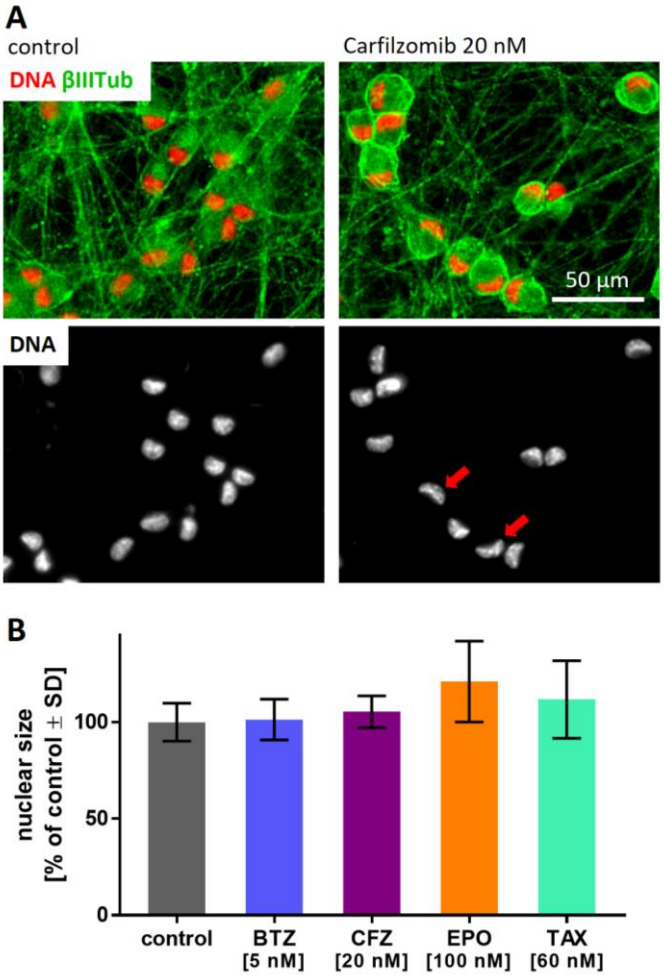

Fig. S11
Holzer et al. 2022

Supplementary Figure S11: Quantifiaction of the nuclear area as cell viability indicator

(A) Sensory neurons were fixed on DoD42 upon exposure to control condition (left) or carfilzomib (20 nM, 24 h) (right). Immunofluorescence images of cells stained for βIII-tubulin (βIIITub) and DNA (using H33342) are shown. DNA staining revealed a deformation of the nuclei in carfilzomib-treated cells, red arrows indicate example nuclei in the single staining image. Images are color coded and the scale bar is given in the images. **(B)** Quantification of the nuclear area of cells cultured under control condition or exposed to bortezomib (BTZ), carfilzomib (CFZ), epoxomicin (EPO) or taxol (TAX) for 24 h at the indicated concentrations. Data are means ± SD of 25 randomly chosen nuclei per condition.

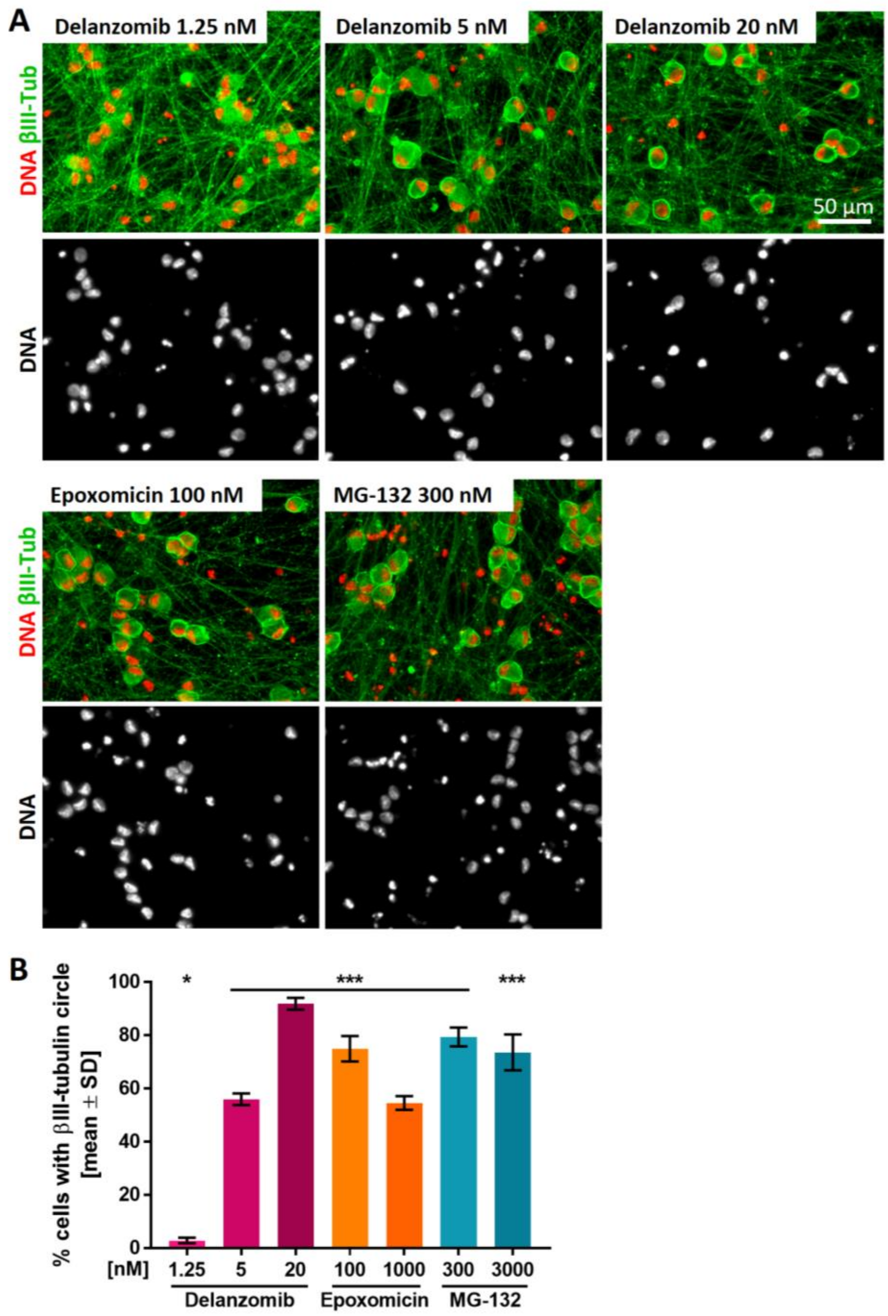

Fig. S12
Holzer et al. 2022

Supplementary Figure S12: Reorganization of the microtubule structure as a general PI effect

Sensory neurons (>DoD38) were exposed to the PIs delanzomib, epoxomicin or MG-132 for 24 h before fixation. Representative immunofluorescence images show stainings of nuclei (DNA, red) and βIII-tubulin (βIIITub, green) (see also figure 7G). Composite images are colour coded and single staining images are shown for cell nuclei (DNA). The scale bar is given in the pictures. **(B)** Quantification of cells exhibiting intense, circular βIII-tubulin staining around the cell somata (covering at least 50% of a full circle). Data are given as % of the total cell count (number of viable cell nuclei) and are means ± SD of 2-3 biological replicates. * $p < 0.05$, *** $p < 0.0001$

Supplementary References

[1] Chen G, Gulbranson DR, Hou Z et al. (2011) Chemically defined conditions for human iPSC derivation and culture. Nat Methods 8, 424–429.

[2] Stiegler NV, Krug AK, Matt F et al. (2011) Assessment of chemical-induced impairment of human neurite outgrowth by multiparametric live cell imaging in high-density cultures. Toxicol Sci 121, 73–87.

[3] Love MI, Huber W, Anders S (2014) Moderated estimation of fold change and dispersion for RNA-seq data with DESeq2. Genome Biol 15, 550.

[4] Karreman C, Klima S, Holzer A-K et al. (2020) CaFFEE: A program for evaluating time courses of Ca2+ dependent signal changes of complex cells loaded with fluorescent indicator dyes. ALTEX 37, 332–336.

[5] Klima S, Brüll M, Spreng A-S et al. (2021) A human stem cell-derived test system for agents modifying neuronal N-methyl-D-aspartate-type glutamate receptor Ca2+-signalling. Arch Toxicol 95, 1703–1722.

[6] Dreser N, Madjar K, Holzer A-K et al. (2020) Development of a neural rosette formation assay (RoFA) to identify neurodevelopmental toxicants and to characterize their transcriptome disturbances. Arch Toxicol 94, 151–171.
